# Low‐cost observations and experiments return a high value in plant phenology research

**DOI:** 10.1002/aps3.11338

**Published:** 2020-04-25

**Authors:** Caitlin McDonough MacKenzie, Amanda S. Gallinat, Lucy Zipf

**Affiliations:** ^1^ Climate Change Institute University of Maine 210 Sawyer Orono Maine 04469 USA; ^2^ Department of Biology Utah State University Logan Utah 84322 USA; ^3^ Ecology Center Utah State University Logan Utah 84322 USA; ^4^ Biology Department Boston University 5 Cummington Mall Boston Massachusetts 02215 USA

**Keywords:** climate change, common garden, natural history, phenology, plant ecology

## Abstract

Plant ecologists in the Anthropocene are tasked with documenting, interpreting, and predicting how plants respond to environmental change. Phenology, the timing of seasonal biological events including leaf‐out, flowering, fruiting, and leaf senescence, is among the most visible and oft‐recorded facets of plant ecology. Climate‐driven shifts in plant phenology can alter reproductive success, interspecific competition, and trophic interactions. Low‐cost phenology research, including observational records and experimental manipulations, is fundamental to our understanding of both the mechanisms and effects of phenological change in plant populations, species, and communities. Traditions of local‐scale botanical phenology observations and data leveraged from written records and natural history collections provide the historical context for recent observations of changing phenologies. New technology facilitates expanding the spatial, taxonomic, and human interest in this research by combining contemporary field observations by researchers and open access community science (e.g., USA National Phenology Network) and available climate data. Established experimental techniques, such as twig cutting and common garden experiments, are low‐cost methods for studying the mechanisms and drivers of plant phenology, enabling researchers to observe phenological responses under novel environmental conditions. We discuss the strengths, limitations, potential hidden costs (i.e., volunteer and student labor), and promise of each of these methods for addressing emerging questions in plant phenology research. Applied thoughtfully, economically, and creatively, many low‐cost approaches offer novel opportunities to fill gaps in our geographic, taxonomic, and mechanistic understanding of plant phenology worldwide.

In the 21st century, a major challenge for plant ecologists is to document, interpret, and predict how plants are responding to rapid environmental changes, such as global land use and climate change (Walther et al., 2002; Thuiller et al., 2008). A complete understanding of changing plant dynamics requires the elucidation of the mechanisms driving changes in plant populations, species, and communities and of the broader implications of those changes for ecosystem processes and patterns. Addressing these challenges can be resource intensive and costly, prohibiting access for many research groups, conservation managers, classrooms, and citizen science initiatives. Meta‐analyses of plant research in herbaria, observational monitoring, and field experiments reveal this disparity at the global scale: research is concentrated in wealthy countries, and plant communities in the Global South are universally underrepresented (Wolkovich et al., 2012; Gill et al., 2015; Daru et al., 2018). It is important to highlight trusted, low‐cost methods in plant ecology to ensure cost does not limit our understanding of plant dynamics and ability to predict future change. Phenology, the study of the timing of seasonal biological events, is both a plant trait and an ecological process. Phenology‐focused research can offer low‐cost options for investigating changes in plant ecology over space and time, as well as for exploring the mechanisms driving those changes and the associated ecological impacts. Here, we review low‐cost options for phenology research that have provided valuable insights into plant responses to environmental change, and that stand to fill remaining gaps with their future use.

Phenology is a highly sensitive indicator of ecological responses to environmental change; it is one of the most visible and oft‐recorded facets of plant ecology (Walther et al., 2002; Cleland et al., 2007). For most plant species, the timing of phenological events is primarily triggered by seasonal climate (Badeck et al., 2004). This, combined with the visibility of plant phenology, has led to its growing global use as an indicator of ecological responses to climate change (Körner and Basler, 2010). Warming‐driven advances in the timing of spring vegetative and reproductive phenology, particularly in temperate ecosystems in the Northern Hemisphere, are now well documented (Parmesan and Yohe, 2003; Cleland et al., 2007; Polgar and Primack, 2011). Plastic phenology has been linked to fitness and demographic stability (Willis et al., 2008; Cleland et al., 2012). These studies report correlations between advancing leaf‐out and flowering and metrics of fitness and persistence. However, recent research determined that in alpine systems, shifts in flowering phenology may be an incomplete and even misleading indicator of population‐level responses to climate change, as early phenology may not compensate for damaging late frosts or increased drought risk during longer growing seasons (Iler et al., 2019). This uncertainty in the relationship between phenological plasticity and fitness hinders attempts to assess climate change vulnerability and distribute limited management resources (Wheatley et al., 2017). Clearly, the field needs further basic research into the mechanisms cueing phenology and the plasticity of phenological responses at the population and species level.

Phenology has broad implications for plant ecology. Flowering and fruiting phenology mediate plant–pollinator interactions, seed dispersal, and reproductive success (Primack, 1987), while leaf‐out and senescence phenology mediate carbon budgets, herbivory, and albedo (Polgar and Primack, 2011; Gallinat et al., 2015). Phenology is linked to multiple intersecting feedback loops from biogeochemical cycles to trophic interactions to local microclimates (Richardson et al., 2013). As environmental changes shift phenology, the effects ripple out across the ecosystem; however, the impacts of changes in phenology are not completely understood and often not incorporated into terrestrial biosphere models (Richardson et al., 2012; Viskari et al., 2015). For example, advancing spring phenology in temperate understory wildflowers is well documented (Miller‐Rushing and Primack, 2008; McDonough MacKenzie et al., 2019), but the concurrent and more rapid advance of canopy leaf‐out has shrunk the window of understory high light levels in early spring, such that carbon budgets for understory wildflowers are likely now smaller than in the 19th century and are predicted to continue to shrink (Heberling et al., 2019). In alpine plant communities, shifts in flowering phenology have shuffled coflowering patterns and redistributed floral abundance across the growing season (CaraDonna et al., 2014). These changing coflowering patterns could affect pollinator foraging activity, pollinator effectiveness, and ultimately reproductive success (Mizunaga and Kudo, 2017; Ramos‐Jiliberto et al., 2018). Much work remains to be done in this field of research, which means that there is abundant low‐hanging fruiting, flowering, and vegetative research amenable to low‐cost investigations into the impacts of phenology on ecosystem processes.

Our knowledge of phenology has been generated through a combination of (1) local‐scale observations, (2) digital expansion to larger spatial scales through natural history collections and citizen science initiatives, and (3) experiments (Fig. 1). Local‐scale observations include the classic records of natural historians and the contemporary researchers who repeat their methods to document phenological shifts at the individual and population level in specific locations. Digital expansion to larger spatial scales leverages the power of both traditional botany (herbaria collections) and 21st‐century naturalists (citizen science platforms such as iNaturalist [https://www.inaturalist.org/]) to compile phenology data across broader spatial, temporal, and taxonomic scales. Experiments, such as common garden and dormant twig manipulations (both highlighted here), relocate plants to new environmental conditions to disentangle the roles of adaptation and environment in phenology. These tools differ in their strengths and limitations (e.g., local‐scale observations clearly describe how wild plant phenology has changed over time, but are limited in generating mechanistic predictions of future change), costs (both explicit and hidden), and potential to address urgent questions about how plants respond to environmental change. These well‐established methods have demonstrated the power of low‐cost phenology research in plant ecology, but a straightforward resource reviewing their strengths, limitations, potential, and relative costs does not yet exist. Here, we do just that; our hope is that by taking stock of what each of these tools has already taught us about plant phenology, we can help direct researchers to the low‐cost tool most appropriate for their research question and available resources (Table 1).

**Figure 1:**
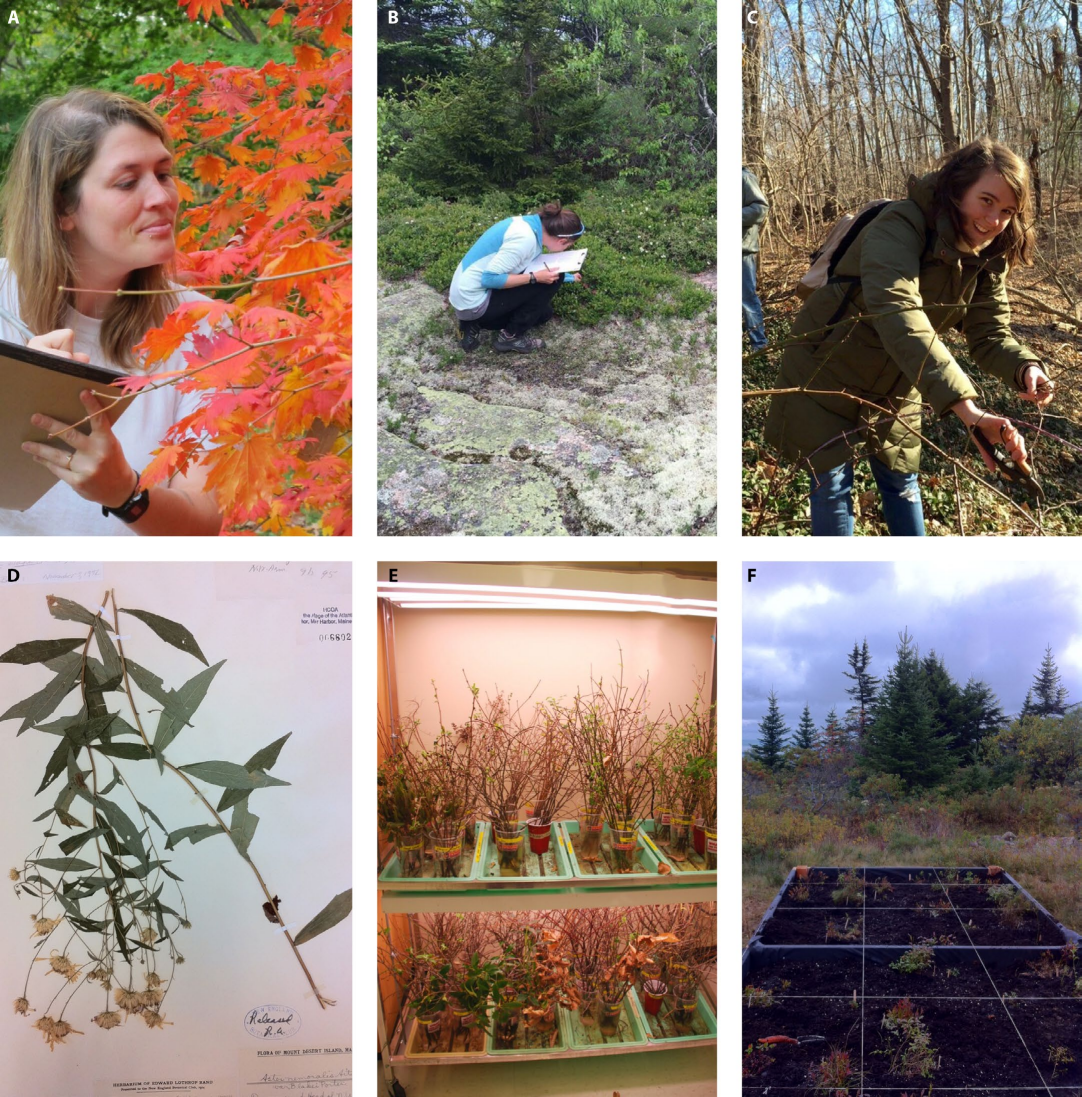
Low‐cost phenology research in action. (A) A.G. records observations of leaf senescence (photo by Richard Primack). (B) C.M.M. records observations of leaf‐out and spring phenology (photo by L.Z.). (C) L.Z. collects dormant twigs for a twig‐cutting experiment (photo by A.G.). (D) Herbarium specimens like this *Aster* sp. contain phenological records (courtesy of College of the Atlantic herbarium, Bar Harbor, Maine, USA). (E) Twig‐cutting experiment with photoperiod treatment in the Primack Lab (photo by A.G.). (F) Raised bed in a reciprocal transplant experiment (photo by C.M.M.).

**Table 1:** The phenology methods outlined in this paper, organized by the types of research questions they best address, resources required, and relative cost of each. The research questions and experimental approaches listed are not exhaustive, but are intended to demonstrate the utility of each method and the value of combining them. Costs for all three approaches can vary widely, but here $ indicates a low cost (e.g., adding phenology observations to an existing study) and $$$$ indicates a high cost (e.g., climate manipulation chambers, which can cost US$10,000–40,000 each).

	**So you want to investigate…**		
	Variation in plant phenology **over time, across species, or with environmental changes in one location**	Variation in plant phenology **across broader geographic regions, species lists, or timescales**	The **mechanisms driving variation** in plant phenology
**Consider using…**	**Local‐scale observations**	**Digital expansion of data**	**Experiments**
**You could address questions like…**	How does the flowering time of *species A* differ across elevations at *site X*? 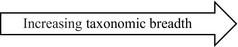	Do close relatives of *species A* have similar elevational sensitivities in their flowering phenology?	**Reciprocal transplants** Is the elevational sensitivity of *species A* due to differences among populations or local environmental conditions?
	Is the order in which species fruit consistent from year to year at *site X*? 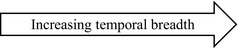	Has the order in which species fruit changed over the past 100 years?	**In situ warming** Are changes in the order of fruiting due to changes in temperature?
	How do canopy trees and herbaceous plants differ in the sensitivity of their leaf‐out times to temperature in *forest X*? 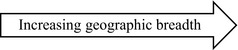	Are differences in leaf‐out sensitivity between canopy trees and herbaceous plants at *site X* similar in other forests around the world?	**Twig experiments** How will the leaf‐out times of different canopy tree species respond to the loss of winter chilling that accompanies spring warming?
**Be prepared to pay for…**	**Person hours** to collect observations	**Person hours** to mine and manage data	**Equipment** to conduct experiments **+** **Person hours** to collect observations
**With a relative cost of…**	**$–$$** Based on duration of study	**$–$$** Based on programming expertise required	**$–$$$$** Cost can vary widely from inexpensive twig experiments to costly in situ warming equipment or climate chambers. For the latter, consider reaching out to **existing experiments** that do not currently monitor phenology

We note that phenology data alone, whether from local‐ or broad‐scale observations or experiments, is not sufficient to answer questions or forecast plant responses to past or future environmental changes. All the low‐cost methods for plant phenology research discussed here, whether they leverage herbarium specimens, transplant gardens, historical observations, or 21st‐century citizen science records, must be supplemented with environmental data. Pairing phenology observations with local climate data allows researchers to answer questions about relationships between phenology and climate over time or across landscapes. Regional weather information can often be freely retrieved online through programs that collate local weather station data across regions or produce satellite‐derived weather products, such as the National Oceanic and Atmospheric Administration's National Centers for Environmental Information's Regional Climate Center Program (https://www.ncei.noaa.gov/) or global products from the Climatic Research Unit (Harris et al., 2014). Mean, minimum, and maximum temperatures are often available for download; however, many research questions may require more site‐specific information (Körner and Hiltbrunner, 2018), in which case temperature loggers (e.g., Onset HOBO brand temperature loggers [UA‐002‐08], US$47 per unit; Onset, Bourne, Massachusetts, USA) must be included in the budget.

## CLASSIC LOCAL‐SCALE OBSERVATIONS

Local‐scale observations, in which phenology is monitored on the ground in one to a few locations and typically by a single research group, have been foundational to our understanding of how phenology varies over time, space, and taxa (Table 1). As evidenced by its long history as a hobby among naturalists, recording local‐scale observations can be an extremely low‐cost strategy for studying plant ecology; these observations require as little as a journal and a writing utensil. Local monitoring can allow researchers to compare phenology among individuals, species, and, if monitoring covers a sufficient environmental gradient across space or time, in changing conditions (McDonough MacKenzie et al., 2019). Historical records, including naturalist, government, and agricultural observations, allow contemporary researchers to measure changes in phenology over dozens to hundreds of years, and have provided a blueprint for how local‐scale phenology monitoring is done (Chuine et al., 2004; Primack et al., 2009; Primack and Miller‐Rushing, 2012; Ellwood et al., 2013). For instance, Henry David Thoreau's records of spring flowering and leaf‐out out in the 1850s, combined with present‐day observations, indicate that many species have advanced their phenology by several weeks (Primack and Miller‐Rushing, 2012; Polgar et al., 2014); observations from cherry blossom festivals in Japan show that cherry trees are now flowering earlier than they have in over 1200 years (Primack et al., 2009); and records from the International Phenological Gardens in Europe from 1959–1996 indicate spring leaf‐out has advanced and autumn leaf senescence has delayed, resulting in a lengthening of the growing season of around 11 days (Menzel, 2000).

Local‐scale observational studies coupled with local meteorological observations can yield suggestions about potential environmental drivers of phenology. In temperate ecosystems, spring phenology (leaf‐out and flowering) is correlated with spring temperature (Walther et al., 2002; Menzel et al., 2006; Cleland et al., 2007), whereas in alpine systems, the timing of snowmelt appears most important (CaraDonna et al., 2014; Theobald et al., 2017; but see Kimball et al., 2014 for an alpine system in the northeastern United States where snowmelt data did not improve model fit for spring phenology in some taxa). The environmental correlates of autumn phenology (fruiting and leaf senescence) have been more challenging to discern, but observational studies point to a combination of temperature, soil moisture, photoperiod, and wind as potential candidates (Gallinat et al., 2015; Gill et al., 2015).

### Strengths of classic local‐scale observations

Classic local‐scale observational studies are powerful. Where historical records exist, they are inexpensive to repeat, and in new locations phenology monitoring can be initiated with little to no investment in equipment (Table 1). Local‐scale observations are well suited to exploring differences in phenology among species; observations of plant communities around the world have indicated that co‐occurring species differ in their spring and autumn phenologies and sensitivities (Polgar and Primack, 2011; Cleland et al., 2012; Fridley, 2012). Botanical gardens can provide particularly species‐rich local‐scale observations; studies of hundreds of species at botanical gardens around the world show striking variation in timing and evolutionary constraints on leaf‐out and fruiting, but not senescence, for temperate woody plants (Panchen et al., 2014, 2015; Gallinat et al., 2018). These community‐wide phenology studies can be used to investigate resource waves, to document the role of phenology in competition and species assembly, and to predict the phenology of additional species using phylogeny. Local observations of wild plant communities are particularly well suited for measurements of intraspecific variation; for instance, Augspurger and Bartlett (2003) showed subcanopy individuals leaf out significantly earlier than canopy individuals of the same species (and experiments later revealed such differences to be ontogenic and not temperature driven; see Vitasse, 2013 and “Experimental Techniques,” below). Comparisons of intra‐ to interspecific variation can tell us more about how plant age, condition, and other qualities interact with external cues.

Local observations are foundational for our understanding of the indirect effects of phenological variation on plant individuals, populations, and communities. For individuals, early leaf‐out and flowering can lead to frost damage (Augspurger, 2009), and long‐term field observations are well positioned to describe the (potentially lagging) fitness consequences of such damage. At the population level, observations of flowering times in Concord, Massachusetts, have shown that taxa that are less responsive to temperature tend to be those with populations in the steepest decline since the 1850s (Willis et al., 2008). Local‐scale observations also offer a window into the community impacts of phenology shifts, such as facilitated invasions, with invasive species occupying parts of the season with relatively low competition but high risk (Wolkovich and Cleland, 2011), benefits conferred to particular functional groups (e.g., trees vs. wildflowers; Heberling et al., 2019), and altered resource availability for other trophic groups (e.g., pollinators; Ogilvie and Thomson, 2015). Because they present the opportunity to deeply investigate a single community, local‐scale observations offer unique advantages for addressing outstanding questions about the indirect effects of phenological change.

### Limitations of classic local‐scale observations

While local‐scale observational studies are straightforward to run, monitoring is time intensive. Although observations across environmental gradients can suggest environmental drivers of phenology, those drivers, in addition to the plastic vs. genetic contributions to phenology, require experimental isolation to confirm. Furthermore, classic observational studies are inherently limited in the transferability of their results across—and ability to make predictions for novel—sites, time frames, and species (Basler, 2016). In part, this is due to modeling limitations; although researchers can model past phenological variation with environmental correlates, the ability of such models to predict future change outside of the range of past variation is often limited. For instance, responses to temperature can be linear below certain warming thresholds, beyond which they can change (Iler et al., 2013). There are statistical solutions for improving models of local‐scale observations to better elucidate relationships with environmental predictors, such as detrending time series phenology data (Iler et al., 2017) and using hierarchical approaches to separate climate from other site‐level differences for better model transferability (Diez et al., 2014).

The difficulty of scaling up and synthesizing local‐scale phenology results can be also be due to data challenges. Meta‐analyses and comparisons across studies may be thwarted by local idiosyncrasies, including monitoring schemes (e.g., Are phenophases recorded for individuals or for a population? Is the intensity of a phenophase, such as peak flowering, recorded or just the date of onset?) and the quality and availability of historical data. These challenges are best addressed by identifying the features important to synthesis and scaling prior to monitoring. This might include reviewing the protocols used in studies with sites, species, and years that could be used for comparison, monitoring the tails and peak of phenophases across a population, or examining the fitness consequences of phenology to estimate broader implications. As we will describe in the following sections, the expansion of phenological observations facilitated by technological advances can increase the transferability of models by providing greater environmental variability and context, while experiments can be used to identify climate thresholds for better predictions. However, it is important to note that once models are generated using these other tools, field‐based observations once again become critical for validating predictions in wild systems (Wolkovich et al., 2012; Elmendorf et al., 2015).

## DIGITAL EXPANSION TO LARGER SPATIAL SCALES

Researchers can leverage broad‐scale, publicly available data to expand the spatial and temporal scale of localized phenology observations (Table 1). Increasing efforts to digitize herbarium specimens, weather data, and field notes have made historical ecological data more widely accessible (Drew et al., 2017), while citizen science programs activate pools of “research assistants” to collect new phenology data via large online networks (Cohn, 2008).

Herbarium records, made up of preserved plant specimens that represent snapshots in time of phenological events, are increasingly used in phenology research, and have expanded researchers’ taxonomic, temporal, and geographic coverage (Willis et al., 2017). Plant specimens with young leaves, visible reproductive structures, or senescing leaves capture the phenological stage of an individual and are kept with a record of the location and date of collection. Specimens can provide phenological information for species and communities well into the past, on the order of 100 years (Primack et al., 2004). The digitization of herbarium records by individual herbaria and larger efforts such as iDigBio (https://www.idigbio.org/portal) have greatly increased the accessibility of these valuable records for use in phenology research (Willis et al., 2017). These online virtual herbaria make specimens available to a wide audience (Soltis, 2017; Canteiro et al., 2019); for example, the iDigBio portal includes almost 20 million plant specimen records.

Data from large‐scale citizen science phenology monitoring programs, which involve interested members of the public in data collection, analysis, or reporting, are another tool researchers can use to expand the spatial and temporal scales of classic observational studies, often at low cost to researchers themselves (Dickinson et al., 2010; Miller‐Rushing et al., 2012; Bonney et al., 2016; Kobori et al., 2016). Citizen science organizations that collect biological observations from people from all backgrounds and experience levels, including the USA National Phenology Network (USA‐NPN; https://www.usanpn.org/), Pl@ntNet (https://plantnet.org/en/), and iNaturalist, are all repositories of phenology data (Kobori et al., 2016). Plant ecologists can incorporate this volunteer‐collected plant phenology data into their projects at no cost (Marchante et al., 2017; Leong and Trautwein, 2019); for example, Nature's Notebook (https://www.usanpn.org/natures_notebook), a program of the USA‐NPN, is a large‐scale phenology monitoring effort in which data is collected by citizen scientists and then made available to researchers (Posthumus and Crimmins, 2011). In 2018, the USA‐NPN had more than 3000 active observers who submitted three million plant and animal phenophase records to their database, contributing to more than 60 peer‐reviewed publications over the past 10 years (USA National Phenology Network, 2018). These large publicly available data sets have enabled researchers to ask questions about species and community phenological change over broad geographic scales (Jeong and Medvigy, 2014; Crimmins et al., 2017) and time periods (Yue et al., 2015).

### Strengths of digital expansion to larger spatial scales

The technology‐facilitated expansion of observational studies can answer research questions at scales that would be difficult for researchers operating at a local scale to address. Digitized specimens, historic data, and citizen science efforts provide a wealth of data to researchers, allowing for the investigation of patterns in environmental drivers of plant phenology over time, across environmental gradients, and among species assemblages at a low cost. Whereas field monitoring studies can be geographically limited, challenging to maintain, and expensive to carry out over many years, long‐term citizen science programs often cover broad geographic areas and many different taxa, and result in large data sets intended for public use (e.g., for climate change research and resource management) (Fuccillo et al., 2015; Bonney et al., 2016). Herbarium specimens are especially valuable in quantifying the effect of climate change on plant species and communities (Calinger et al., 2013; Davis et al., 2015), and offer further opportunities to fill taxonomic (e.g., grasses; Primack and Gallinat, 2017) and regional (e.g., the tropics; Zalamea et al., 2011) gaps in this field. This increased breadth improves our ability to test phenological predictions born from local‐scale observations and experimental manipulations across sites.

### Limitations of digital expansion to larger spatial scales

Like classic observational studies, volunteer data‐driven projects investigate variation in phenology across communities, time, and space, but are limited in their capacity to demonstrate mechanisms underlying ecological patterns beyond environmental correlates. The lack of standardization in phenological scoring across studies that employ herbarium specimens has made it difficult to combine published data with field observations (Soltis, 2017; Pearson, 2019); however, efforts to generate standards for scoring specimens (Yost et al., 2018) and synthesize data collected with different methods using shared language (Stucky et al., 2018) further indicate the growing potential of this widespread data source.

There are also limits and sources of error associated with volunteer‐collected data. Phenology observations gathered by organizations such as Nature's Notebook and iNaturalist are not collected with a specific research question in mind, which can limit the number and type of research questions citizen science data can help answer. However, recent research provides guidelines for employing crowd‐sourced phenology monitoring data sets (Crimmins et al., 2017; Taylor, 2019); for example, geographic scale is an important factor in selecting volunteer‐collected data to include in phenology studies. Smaller site‐ to landscape‐scale studies of plant phenology benefit from more conservative data selection criteria that have less potential for error than do larger‐scale projects (Gerst et al., 2016; Taylor et al., 2019). Involving communities in data collection also often introduces biases into studies; for instance, citizen scientists favor charismatic locations and easy‐to‐identify species, excluding rare or hard‐to‐find species and study systems (McDonough MacKenzie et al., 2017). These limitations should urge researchers to assess citizen science data individually for quality and applicability to their research question (Fuccillo et al., 2015; Kosmala et al., 2016). However, the substantial body of literature that successfully utilizes citizen science data to answer ecological questions indicates the existence of many high‐quality citizen science data sets with important scientific potential.

## EXPERIMENTAL TECHNIQUES

In addition to carrying out low‐cost observations of phenology, researchers can employ cost‐effective methods to experimentally manipulate phenology under defined conditions (Table 1). These experimental manipulations can range from simple twig cuttings to more resource‐intensive, but still low‐cost, common gardens. Relocating either parts of plants or entire plants in experiments reveals phenological reaction norms under different, and sometimes novel, environmental conditions and can uncover the mechanisms of observed patterns (Wolkovich et al., 2012; Berend et al., 2019). Similarly, phenology can be monitored following the manipulation of environmental conditions in the field with low‐tech experiments such as open‐topped chambers (Welshofer et al., 2017) or snow removal (Sorensen et al., 2016), without relocating plants. Experimental manipulations can be especially powerful in the context of environmental change research because they can be designed to match future climate forecasts. Thus, experiments allow researchers to test hypotheses that studies using observational findings may only be able to approach opportunistically.

Here, we highlight two examples of low‐cost phenology experiments: twig cuttings and transplants. Both cuttings and transplants fall under the broad umbrella of common garden studies, experimental manipulations that place specimens from different provenances in common environmental conditions (Clausen et al., 1947). These common garden studies differentiate local adaptation from phenotypic plasticity: when individuals or twigs are exposed to common conditions, differences in phenotypes must represent differences in genotypes. Implementing these low‐cost experiments is straightforward. Experiments using twig cuttings involve collecting dormant twigs from the field and monitoring phenology in the lab (Polgar et al., 2014; Primack et al., 2015). Although some manipulations (e.g., humidity [Laube et al., 2014; but see Zipf and Primack, 2017] and freeze–thaw treatments [Muffler et al., 2016; but see Bielenberg and Gasic, 2019]) require specialized growth chambers, winter twig cuttings exposed to warmer, indoor temperatures and/or light treatments are low cost and low maintenance (Primack et al., 2015). Transplant experiments are more resource intensive than twig cutting, but can still be designed at low cost. Seeds, seedlings, or mature plants may be transplanted from their provenance into a garden that could be as simple as a plot in the ground (Parker et al., 2017), or as high‐tech (and high‐cost) as a specialized growth chamber (Rossi and Isabel, 2016). Between these extremes are many low‐cost forcing options for transplants grown in greenhouses (Wadgymar et al., 2018a), raised beds (McDonough MacKenzie et al., 2018), or in local soil (Hamann et al., 2017). The transplant design depends on the specific research questions of the study (see Berend et al., 2019 for a review of different transplant designs).

### Strengths of experimental techniques

Twig cuttings in particular are well suited to questions about the mechanistic drivers of phenology; simple manipulations of winter chilling, day length, and spring warming have the potential to fill gaps in our basic understanding of variations in species‐ and population‐level phenology (Körner and Basler, 2010). Twig‐cutting experiments have exposed how lower to nonexistent chilling requirements in nonnative trees and shrubs allow these taxa to leaf out much earlier than native trees and shrubs in warmer springs (Polgar et al., 2014). Although isolating potential phenology drivers in the field using transplants is more challenging than with twig cuttings, comparing environmental conditions between transplant gardens, or adding experimental treatments to gardens, such as watering, allows researchers to test the relative importance of different mechanisms that may be driving phenological responses (Alexander, 2016; Kueppers et al., 2017). Transplant experiments can also be leveraged to test the ecological effects of phenological shifts, for example, incorporating measurements of competition and phenological synchrony with local populations (Alexander et al., 2015).

### Limitations of experimental techniques

The experimental designs for both twig cutting and transplants are limited by a lack of standardization, which makes meta‐analyses and comparisons across studies extremely challenging (Berend et al., 2019). Experimental manipulations have been shown to underestimate shifts in leaf‐out and flowering phenology compared to observational monitoring records (Wolkovich et al., 2012). Twig cuttings are further limited because they are just a small part of a larger organism; this makes the method far more reliable for studies of leaf‐out phenology than for flowering and leaf senescence (Primack et al., 2015). Similarly, transplant experiments are limited to taxa that can reasonably be transplanted (i.e., small shrubs and herbaceous species or seedlings of trees). These limitations are important as phenology varies with ontogeny (Osada et al., 2002; Yang and Rudolf, 2010). Furthermore, as discussed above, experiments that alter environmental variables can quickly become expensive and high maintenance when treatments such as drought, frost events, and humidity are incorporated into the experimental design (Laube et al., 2014; Gugger et al., 2015; Muffler et al., 2016). While all of these environmental conditions are important to consider, there are explicit gaps in our basic knowledge of phenology that low‐cost experiments can address with simple experimental designs.

## COMBINED TECHNIQUES FOR GREATER INSIGHT

Matching experiments with local‐ and regional‐scale observations makes it possible to explore new research questions around the relationships between phenology, reproductive success, fitness, and population persistence. Local‐scale observational studies can be combined with both herbarium specimens, to increase temporal resolution (Rivera and Borchert, 2001; Panchen et al., 2012), and with citizen science observations, to both expand study extent and validate crowd‐sourced data quality (Kosmala et al., 2016). Studies combining phenology experiments and local observations are especially relevant for management and climate change vulnerability assessments; for example, long‐term observational records and a low‐tech snow‐removal experiment at Rocky Mountain Biological Station, Colorado, USA, revealed that climate change is decoupling the historical combinations of photoperiod and spring temperatures that cue the onset of flowering for subalpine species (Wadgymar et al., 2018b). These changing climatic drivers of phenology can reduce the probability of flowering for plant species and depress their fitness. In Acadia National Park, Maine, USA, observational transects and reciprocal transplants were used to confirm that leaf‐out phenology in three understory taxa is plastic (McDonough MacKenzie et al., 2018, 2019). The local environment, not genetic adaptation, drives intraspecific variation in leaf‐out along elevation gradients that climb from temperate deciduous forests to open subalpine ridges, which may indicate that populations across these microclimates are resilient to climate change.

## CONCLUSIONS

Monitoring plant phenology over time, space, species, and experimental manipulations can characterize change as well as reveal the mechanisms behind the observed changes. Each of the low‐cost tools described here for phenology research have unique strengths and limitations. Local‐scale observations are powerful for describing what is happening, particularly in the wild, and are best used to measure variation over time, space, and species. Coupled with environmental observations, local‐scale observations can even suggest what might be driving phenological variation for particular plant populations or communities, but can be limited in their ability to identify phenological mechanisms and generate predictions over species, space, and time. Expanding the taxonomic and geographic breadth of local‐scale observations with technological applications and citizen science can improve the transferability of phenology models across space and species, and can have the added advantage of expanding community engagement (Bonney et al., 2016). However, this option can require substantial time and energy for training, volunteer engagement, and data management (Wiggins, 2013). Experiments, meanwhile, can be used to explicitly test the mechanisms driving phenological variation and improve predictions for novel conditions, but they can range in cost, and may not accurately predict the magnitude of responses in wild plants (Wolkovich et al., 2012). Thus, it is not surprising that these three low‐cost phenology tools provide the most information when used in synthesis with one another. Local‐scale observations provide a foundation for identifying which mechanisms should be tested with experiments, and experimental models should be validated with local‐scale observations to ensure models reflect wild plant responses. Validating models across a broader range of species and sites using tech‐enabled data sets ensures greater model transferability.

These techniques are poised to address some of the most conspicuous gaps in phenology research. Gaps in phenological data are both geographic and taxonomic, including non‐temperate and Southern Hemisphere ecosystems, and plant taxa that have been traditionally overlooked by phenology researchers (e.g., grasses and evergreens). Researchers working in these regions and clades are well positioned to use any (or all) of these low‐cost tools to identify how phenology is changing for populations, species, and communities (using local‐scale or tech‐enabled observations), what the mechanisms behind those changes are (by coupling observations with meteorological data, or with experiments), or how they compare to other sites and species (using the growing availability of tech‐enabled observations). All three of these tools are critical for addressing the gaps in *explaining* and *predicting* variation in phenology for use in conservation and resource management. Local‐scale observations, particularly coupled with experiments to investigate potential responses to novel conditions, will be of particular value to local‐scale conservation, and will be even more valuable when considering local community‐scale impacts (e.g., predicting shifts in local plant phenology, and estimating future overlap with target pollinators). Beyond these local predictions, it is increasingly important for broad‐scale ecological efforts that models be transferable across systems (Peters, 1991). Indeed, one of the best methods the phenology research community can employ now for reducing monitoring costs in the future (by reducing the need for additional independent programs observing local‐scale phenology) is to leverage the broad‐scale data sets that have been developed following recent technological advances to describe and predict broad‐scale heterogeneity. Using ecological forecasting techniques, researchers can estimate uncertainty across space and time and iteratively improve phenological predictions in the near term for faster management responses (Dietze et al., 2018).

Many applications of the techniques we have described here require little monetary investment (see Fitchett et al., 2015 for a description of the range of phenology tools available regardless of cost). Phenology monitoring projects often rely on monitoring by volunteers and students to operate at extremely low costs (Koch et al., 2007), and while experiments can also cover a broad range of equipment costs, all three of the tools detailed above share explicit and hidden costs in the form of researchers’ time. We caution phenology researchers to remember that low‐cost does not mean no‐cost, namely when considering person‐hours. In fact, researchers have a golden opportunity in phenology research to use the affordability of the tools themselves to include more paid opportunities for participants. Money not spent on equipment or collecting across a wide range of sites—thanks to tech‐enabled data sharing that allows researchers to generate geographic context with an internet connection—can be used to generate paying positions, so that the phenology research is not dependent on unpaid volunteers (who may well be available), because relying on underpaid students and researchers who can work in exchange for research credit or experience can create barriers to entry rooted in wealth and privilege (Fournier and Bond, 2015). Phenological research, like all science, benefits from inclusion and a diversity of life experiences (Nature, 2018).

## References

[aps311338-bib-0001] Alexander, J. M. 2016 Experiments link competition and climate change responses. Journal of Vegetation Science 27(2): 217–218.

[aps311338-bib-0002] Alexander, J. M. , J. M. Diez , and J. M. Levine . 2015 Novel competitors shape species’ responses to climate change. Nature 525: 515–518. 26374998 10.1038/nature14952

[aps311338-bib-0003] Augspurger, C. K. 2009 Spring 2007 warmth and frost: Phenology, damage and refoliation in a temperate deciduous forest. Functional Ecology 23: 1031–1039.

[aps311338-bib-0004] Augspurger, C. K. , and E. A. Bartlett . 2003 Differences in leaf phenology between juvenile and adult trees in a temperate deciduous forest. Tree Physiology 23: 517–525. 12730043 10.1093/treephys/23.8.517

[aps311338-bib-0005] Badeck, F. W. , A. Bondeau , K. Böttcher , D. Doktor , W. Lucht , J. Schaber , and S. Sitch . 2004 Responses of spring phenology to climate change. New Phytologist 162: 295–309.

[aps311338-bib-0006] Basler, D. 2016 Evaluating phenological models for the prediction of leaf‐out dates in six temperate tree species across central Europe. Agricultural and Forest Meteorology 217: 10–21.

[aps311338-bib-0007] Berend, K. , K. Haynes , and C. McDonough MacKenzie . 2019 Common garden experiments as a dynamic tool for ecological studies of alpine plants and communities in northeastern North America. Rhodora 121(987): 174–212.

[aps311338-bib-0008] Bielenberg, D. G. , and K. Gasic . 2019 Controlled‐temperature treatments with low‐cost, off‐the‐shelf equipment for bud or seed forcing experiments. HortScience 54: 766–768.

[aps311338-bib-0009] Bonney, R. , T. B. Phillips , H. L. Ballard , and J. W. Enck . 2016 Can citizen science enhance public understanding of science? Public Understanding of Science 25: 2–16. 26445860 10.1177/0963662515607406

[aps311338-bib-0010] Calinger, K. M. , S. Queenborough , and P. S. Curtis . 2013 Herbarium specimens reveal the footprint of climate change on flowering trends across north‐central North America. Ecology Letters 16: 1037–1044. 23786499 10.1111/ele.12135 PMC3806244

[aps311338-bib-0011] Canteiro, C. , L. Barcelos , F. Filardi , R. Forzza , L. Green , J. Lanna , P. Leitman , et al. 2019 Enhancement of conservation knowledge through increased access to botanical information. Conservation Biology 33: 523–533. 30809858 10.1111/cobi.13291 PMC6850347

[aps311338-bib-0012] CaraDonna, P. J. , A. M. Iler , and D. W. Inouye . 2014 Shifts in flowering phenology reshape a subalpine plant community. Proceedings of the National Academy of Sciences USA 111: 4916–4921. 10.1073/pnas.1323073111 PMC3977233 24639544

[aps311338-bib-0013] Chuine, I. , P. Yiou , N. Viovy , B. Seguin , V. Daux , and E. L. R. Ladurie . 2004 Grape ripening as a past climate indicator. Nature 432: 289–290. 15549085 10.1038/432289a

[aps311338-bib-0014] Clausen, J. , D. D. Keck , and W. M. Hiesey . 1947 Heredity of geographically and ecologically isolated races. American Naturalist 81: 114–143. 10.1086/281507 20297036

[aps311338-bib-0015] Cleland, E. E. , I. Chuine , A. Menzel , H. A. Mooney , and M. D. Schwartz . 2007 Shifting plant phenology in response to global change. Trends in Ecology and Evolution 22: 357–365. 17478009 10.1016/j.tree.2007.04.003

[aps311338-bib-0016] Cleland, E. E. , J. M. Allen , T. M. Crimmins , J. A. Dunne , S. Pau , S. E. Travers , E. S. Zavaleta , and E. M. Wolkovich . 2012 Phenological tracking enables positive species responses to climate change. Ecology 93: 1765–1771. 22928404 10.1890/11-1912.1

[aps311338-bib-0017] Cohn, J. P. 2008 Citizen science: Can volunteers do real research? BioScience 58: 192–197.

[aps311338-bib-0018] Crimmins, T. M. , M. A. Crimmins , K. L. Gerst , A. H. Rosemartin , and J. F. Weltzin . 2017 USA National Phenology Network's volunteer‐contributed observations yield predictive models of phenological transitions. PLoS ONE 12: e0182919. 28829783 10.1371/journal.pone.0182919 PMC5568737

[aps311338-bib-0019] Daru, B. H. , D. S. Park , R. B. Primack , C. G. Willis , D. S. Barrington , T. J. Whitfeld , T. G. Seidler , et al. 2018 Widespread sampling biases in herbaria revealed from large‐scale digitization. New Phytologist 217: 939–955. 29083043 10.1111/nph.14855

[aps311338-bib-0020] Davis, C. C. , C. G. Willis , B. Connolly , C. Kelly , and A. M. Ellison . 2015 Herbarium records are reliable sources of phenological change driven by climate and provide novel insights into species’ phenological cueing mechanisms. American Journal of Botany 102: 1599–1609. 26451038 10.3732/ajb.1500237

[aps311338-bib-0021] Dickinson, J. L. , B. Zuckerberg , and D. N. Bonter . 2010 Citizen science as an ecological research tool: Challenges and benefits. Annual Review of Ecology, Evolution and Systematics 41: 149–172.

[aps311338-bib-0022] Dietze, M. C. , A. Fox , L. M. Beck‐Johnson , J. L. Betancourt , M. B. Hooten , C. S. Jarnevich , T. H. Keitt , et al. 2018 Iterative near‐term ecological forecasting: Needs, opportunities, and challenges. Proceedings of the National Academy of Sciences USA 115: 1424–1432. 10.1073/pnas.1710231115 PMC5816139 29382745

[aps311338-bib-0023] Diez, J. M. , I. Ibáñez , J. A. Silander Jr. , R. Primack , H. Higuchi , H. Kobori , A. Sen , and T. Y. James . 2014 Beyond seasonal climate: Statistical estimation of phenological responses to weather. Ecological Applications 24(7): 1793–1802. 29210238 10.1890/13-1533.1

[aps311338-bib-0024] Drew, J. A. , C. S. Moreau , and M. L. J. Stiassny . 2017 Digitization of museum collections holds the potential to enhance researcher diversity. Nature Ecology and Evolution 1: 1789–1790. 29133899 10.1038/s41559-017-0401-6

[aps311338-bib-0025] Ellwood, E. R. , S. A. Temple , R. B. Primack , N. L. Bradley , and C. C. Davis . 2013 Record‐breaking early flowering in the Eastern United States. PLoS ONE 8: e53788. 23342001 10.1371/journal.pone.0053788 PMC3547064

[aps311338-bib-0026] Elmendorf, S. C. , G. H. Henry , R. D. Hollister , A. M. Fosaa , W. A. Gould , L. Hermanutz , A. Hofgaard , et al. 2015 Experiment, monitoring, and gradient methods used to infer climate change effects on plant communities yield consistent patterns. Proceedings of the National Academy of Sciences USA 112(2): 448–452. 10.1073/pnas.1410088112 PMC4299205 25548195

[aps311338-bib-0027] Fitchett, J. M. , S. W. Grab , and D. I. Thompson . 2015 Plant phenology and climate change: Progress in methodological approaches and application. Progress in Physical Geography 39(4): 460–482.

[aps311338-bib-0028] Fournier, A. M. V. , and A. L. Bond . 2015 Volunteer field technicians are bad for wildlife ecology. Wildlife Society Bulletin 39: 819–821.

[aps311338-bib-0029] Fridley, J. D. 2012 Extended leaf phenology and the autumn niche in deciduous forest invasions. Nature 485: 359–362. 22535249 10.1038/nature11056

[aps311338-bib-0030] Fuccillo, K. K. , T. M. Crimmins , C. E. de Rivera , and T. S. Elder . 2015 Assessing accuracy in citizen science‐based plant phenology monitoring. International Journal of Biometeorology 59: 917–926. 25179528 10.1007/s00484-014-0892-7

[aps311338-bib-0031] Gallinat, A. S. , R. B. Primack , and D. L. Wagner . 2015 Autumn, the neglected season in climate change research. Trends in Ecology and Evolution 30: 169–176. 25662784 10.1016/j.tree.2015.01.004

[aps311338-bib-0032] Gallinat, A. S. , R. B. Primack , C. G. Willis , B. Nordt , A. D. Stevens , R. Fahey , A. T. Whittemore , et al. 2018 Patterns and predictors of fleshy fruit phenology at five international botanical gardens. American Journal of Botany 105: 1824–1834. 30418679 10.1002/ajb2.1189

[aps311338-bib-0033] Gerst, K. L. , J. L. Kellermann , C. A. F. Enquist , A. H. Rosemartin , and E. G. Denny . 2016 Estimating the onset of spring from a complex phenology database: Trade‐offs across geographic scales. International Journal of Biometeorology 60: 391–400. 26260630 10.1007/s00484-015-1036-4

[aps311338-bib-0034] Gill, A. L. , A. S. Gallinat , R. Sanders‐DeMott , A. J. Rigden , D. J. Short Gianotti , J. A. Mantooth , and P. H. Templer . 2015 Changes in autumn senescence in northern hemisphere deciduous trees: A meta‐analysis of autumn phenology studies. Annals of Botany 116: 875–888. 25968905 10.1093/aob/mcv055 PMC4640124

[aps311338-bib-0035] Gugger, S. , H. Kesselring , J. Stöcklin , and E. Hamann . 2015 Lower plasticity exhibited by high‐ versus mid‐elevation species in their phenological responses to manipulated temperature and drought. Annals of Botany 116: 953–962. 26424784 10.1093/aob/mcv155 PMC4640129

[aps311338-bib-0036] Hamann, E. , J. F. Scheepens , H. Kesselring , G. F. J. Armbruster , and J. Stöcklin . 2017 High intraspecific phenotypic variation, but little evidence for local adaptation in *Geum reptans* populations in the Central Swiss Alps. Alpine Botany 127: 121–132.

[aps311338-bib-0037] Harris, I. P. D. J. , P. D. Jones , T. J. Osborn , and D. H. Lister . 2014 Updated high‐resolution grids of monthly climatic observations–the CRU TS3.10. Dataset. International Journal of Climatology 34(3): 623–642.

[aps311338-bib-0038] Heberling, J. M. , C. McDonough MacKenzie , J. D. Fridley , S. Kalisz , and R. B. Primack . 2019 Phenological mismatch with trees reduces wildflower carbon budgets. Ecology Letters 22: 616–623. 30714287 10.1111/ele.13224

[aps311338-bib-0039] Iler, A. M. , T. T. Høye , D. W. Inouye , and N. M. Schmidt . 2013 Nonlinear flowering responses to climate: Are species approaching their limits of phenological change? Philosophical Transactions of the Royal Society B: Biological Sciences 368(1624): 20120489. 10.1098/rstb.2012.0489 PMC3720060 23836793

[aps311338-bib-0040] Iler, A. M. , D. W. Inouye , N. M. Schmidt , and T. T. Høye . 2017 Detrending phenological time series improves climate‐phenology analyses and reveals evidence of plasticity. Ecology 98: 647–655. 27984645 10.1002/ecy.1690

[aps311338-bib-0041] Iler, A. M. , A. Compagnoni , D. W. Inouye , J. L. Williams , P. J. CaraDonna , A. Anderson , and T. E. X. Miller . 2019 Reproductive losses due to climate change‐induced earlier flowering are not the primary threat to plant population viability in a perennial herb. Journal of Ecology 107: 1931–1943.

[aps311338-bib-0042] Jeong, S. J. , and D. Medvigy . 2014 Macroscale prediction of autumn leaf coloration throughout the continental United States. Global Ecology and Biogeography 23: 1245–1254.

[aps311338-bib-0043] Kimball, K. D. , M. L. Davis , D. M. Weihrauch , G. L. D. Murray , and K. Rancourt . 2014 Limited alpine climatic warming and modeled phenology advancement for three alpine species in the Northeast United States. American Journal of Botany 101: 1437–1446. 25253704 10.3732/ajb.1400214

[aps311338-bib-0044] Kobori, H. , J. L. Dickinson , I. Washitani , R. Sakurai , T. Amano , N. Komatsu , W. Kitamura , et al. 2016 Citizen science: A new approach to advance ecology, education, and conservation. Ecological Research 31: 1–19.

[aps311338-bib-0045] Koch, E. , E. Bruns , F. M. Chmielewski , C. Defila , W. Lipa , and A. Menzel . 2007 World Climate Data and Monitoring Programme: Guidelines for plant phenological observations. World Meteorological Organization, Geneva, Switzerland Available at https://library.wmo.int/doc_num.php?explnum_id=9414 [accessed 11 March 2020].

[aps311338-bib-0046] Körner, C. , and D. Basler . 2010 Phenology under global warming. Science 327: 1461–1462. 20299580 10.1126/science.1186473

[aps311338-bib-0047] Körner, C. , and E. Hiltbrunner . 2018 The 90 ways to describe plant temperature. Perspectives in Plant Ecology, Evolution and Systematics 30: 16–21.

[aps311338-bib-0048] Kosmala, M. , A. Wiggins , A. Swanson , and B. Simmons . 2016 Assessing data quality in citizen science. Frontiers in Ecology and the Environment 14: 551–560.

[aps311338-bib-0049] Kueppers, L. M. , E. Conlisk , C. Castanha , A. B. Moyes , M. J. Germino , P. de Valpine , M. S. Torn , and J. B. Mitton . 2017 Warming and provenance limit tree recruitment across and beyond the elevation range of subalpine forest. Global Change Biology 23: 2383–2395. 27976819 10.1111/gcb.13561

[aps311338-bib-0050] Laube, J. , T. H. Sparks , N. Estrella , and A. Menzel . 2014 Does humidity trigger tree phenology? Proposal for an air humidity based framework for bud development in spring. New Phytologist 202: 350–355. 24404784 10.1111/nph.12680

[aps311338-bib-0051] Leong, M. , and M. Trautwein . 2019 A citizen science approach to evaluating US cities for biotic homogenization. PeerJ 7: e6879. 31106074 10.7717/peerj.6879 PMC6499060

[aps311338-bib-0052] Marchante, H. , M. C. Morais , A. Gamela , and E. Marchante . 2017 Using a webmapping platform to engage volunteers to collect data on invasive plants distribution. Transactions in GIS 21: 238–252.

[aps311338-bib-0053] McDonough MacKenzie, C. , G. Murray , R. Primack , and D. Weihrauch . 2017 Lessons from citizen science: Assessing volunteer‐collected plant phenology data with Mountain Watch. Biological Conservation 208: 121–126.

[aps311338-bib-0054] McDonough MacKenzie, C. , R. B. Primack , and A. J. Miller‐Rushing . 2018 Local environment, not local adaptation, drives leaf‐out phenology in common gardens along an elevational gradient in Acadia National Park, Maine. American Journal of Botany 105: 986–995. 29957884 10.1002/ajb2.1108

[aps311338-bib-0055] McDonough MacKenzie, C. M. , R. B. Primack , and A. J. Miller‐Rushing . 2019 Trails‐as‐transects: Phenology monitoring across heterogeneous microclimates in Acadia National Park, Maine. Ecosphere 10(3): e02626.

[aps311338-bib-0056] Menzel, A. 2000 Trends in phenological phases in Europe between 1951 and 1996. International Journal of Biometeorology 44: 76–81. 10993561 10.1007/s004840000054

[aps311338-bib-0057] Menzel, A. , T. H. Sparks , N. Estrella , E. Koch , A. Aaasa , R. Ahas , K. Alm‐Kübler , et al. 2006 European phenological response to climate change matches the warming pattern. Global Change Biology 12: 1969–1976.

[aps311338-bib-0058] Miller‐Rushing, A. J. , and R. B. Primack . 2008 Global warming and flowering times in Thoreau's Concord: A community perspective. Ecology 89(2): 332–341. 18409423 10.1890/07-0068.1

[aps311338-bib-0059] Miller‐Rushing, A. , R. Primack , and R. Bonney . 2012 The history of public participation in ecological research. Frontiers in Ecology and the Environment 10: 285–290.

[aps311338-bib-0060] Mizunaga, Y. , and G. Kudo . 2017 A linkage between flowering phenology and fruit‐set success of alpine plant communities with reference to the seasonality and pollination effectiveness of bees and flies. Oecologia 185: 453–464. 28889327 10.1007/s00442-017-3946-9

[aps311338-bib-0061] Muffler, L. , C. Beierkuhnlein , G. Aas , A. Jentsch , A. H. Schweiger , C. Zohner , and J. Kreyling . 2016 Distribution ranges and spring phenology explain late frost sensitivity in 170 woody plants from the Northern Hemisphere. Global Ecology and Biogeography 25: 1061–1071.

[aps311338-bib-0062] Nature . 2018 Science benefits from diversity. Nature 558: 5. 10.1038/d41586-018-05326-3 31076730

[aps311338-bib-0063] Ogilvie, J. E. , and J. D. Thomson . 2015 Male bumble bees are important pollinators of a late‐blooming plant. Arthropod‐Plant Interactions 9: 205–213.

[aps311338-bib-0064] Osada, N. , H. Takeda , A. Furukawa , and M. Awang . 2002 Ontogenetic changes in leaf phenology of a canopy species, *Elateriospermum tapos* (Euphorbiaceae), in a Malaysian rain forest. Journal of Tropical Ecology 18: 91–105.

[aps311338-bib-0065] Panchen, Z. A. , R. B. Primack , T. Aniśko , and R. E. Lyons . 2012 Herbarium specimens, photographs, and field observations show Philadelphia area plants are responding to climate change. American Journal of Botany 99: 751–756. 22447982 10.3732/ajb.1100198

[aps311338-bib-0066] Panchen, Z. A. , R. B. Primack , B. Nordt , E. R. Ellwood , A. D. Stevens , S. S. Renner , C. G. Willis , et al. 2014 Leaf out times of temperate woody plants are related to phylogeny, deciduousness, growth habit and wood anatomy. New Phytologist 203: 1208–1219. 24942252 10.1111/nph.12892

[aps311338-bib-0067] Panchen, Z. A. , R. B. Primack , A. S. Gallinat , B. Nordt , A. D. Stevens , Y. Du , and R. Fahey . 2015 Substantial variation in leaf senescence times among 1360 temperate woody plant species: Implications for phenology and ecosystem processes. Annals of Botany 116: 865–873. 25808654 10.1093/aob/mcv015 PMC4640117

[aps311338-bib-0068] Parker, T. C. , J. Tang , M. B. Clark , M. M. Moody , and N. Fetcher . 2017 Ecotypic differences in the phenology of the tundra species *Eriophorum vaginatum* reflect sites of origin. Ecology and Evolution 69: 491–512. 10.1002/ece3.3445 PMC5696421 29188008

[aps311338-bib-0069] Parmesan, C. , and G. Yohe . 2003 A globally coherent fingerprint of climate change impacts across natural systems. Nature 421: 37–42. 12511946 10.1038/nature01286

[aps311338-bib-0070] Pearson, K. D. 2019 A new method and insights for estimating phenological events from herbarium specimens. Applications in Plant Sciences 7(3): e01224. 30937217 10.1002/aps3.1224 PMC6426155

[aps311338-bib-0071] Peters, R. H. 1991 A critique for ecology. Cambridge University Press, Cambridge, United Kingdom.

[aps311338-bib-0072] Polgar, C. A. , and R. B. Primack . 2011 Leaf‐out phenology of temperate woody plants: From trees to ecosystems. New Phytologist 191: 926–941. 21762163 10.1111/j.1469-8137.2011.03803.x

[aps311338-bib-0073] Polgar, C. , A. Gallinat , and R. B. Primack . 2014 Drivers of leaf‐out phenology and their implications for species invasions: Insights from Thoreau's Concord. New Phytologist 202: 106–115. 24372373 10.1111/nph.12647

[aps311338-bib-0074] Posthumus, E. , and T. Crimmins . 2011 Nature's Notebook: A tool for education and research. Bulletin of the Ecological Society of America 92: 185–187.

[aps311338-bib-0075] Primack, R. B. 1987 Relationships among flowers, fruits, and seeds. Annual Review of Ecology and Systematics 18: 409–430.

[aps311338-bib-0076] Primack, R. B. , and A. S. Gallinat . 2017 Insights into grass phenology from herbarium specimens. New Phytologist 213: 1567–1568. 28164336 10.1111/nph.14439

[aps311338-bib-0077] Primack, R. B. , and A. J. Miller‐Rushing . 2012 Uncovering, collecting, and analyzing records to investigate the ecological impacts of climate change: A template from Thoreau's Concord. BioScience 62: 170–181.

[aps311338-bib-0078] Primack, D. , C. Imbres , R. B. Primack , A. J. Miller‐Rushing , and P. Del Tredici . 2004 Herbarium specimens demonstrate earlier flowering times in response to warming in Boston. American Journal of Botany 91: 1260–1264. 21653483 10.3732/ajb.91.8.1260

[aps311338-bib-0079] Primack, R. B. , H. Higuchi , and A. J. Miller‐Rushing . 2009 The impact of climate change on cherry trees and other species in Japan. Biological Conservation 142: 1943–1949.

[aps311338-bib-0080] Primack, R. B. , J. Laube , A. S. Gallinat , and A. Menzel . 2015 From observations to experiments in phenology research: Investigating climate change impacts on trees and shrubs using dormant twigs. Annals of Botany 116: 889–897. 25851135 10.1093/aob/mcv032 PMC4640118

[aps311338-bib-0081] Ramos‐Jiliberto, R. , P. Moisset de Espanés , M. Franco‐Cisterna , T. Petanidou , and D. P. Vázquez . 2018 Phenology determines the robustness of plant–pollinator networks. Scientific Reports 8: 14873. 30291278 10.1038/s41598-018-33265-6 PMC6173761

[aps311338-bib-0082] Richardson, A. D. , R. S. Anderson , M. A. Arain , A. G. Barr , G. Bohrer , G. Chen , J. M. Chen , et al. 2012 Terrestrial biosphere models need better representation of vegetation phenology: Results from the North American Carbon Program Site Synthesis. Global Change Biology 18: 566–584.

[aps311338-bib-0083] Richardson, A. D. , T. F. Keenan , M. Migliavacca , Y. Ryu , O. Sonnentag , and M. Toomey . 2013 Climate change, phenology, and phenological control of vegetation feedbacks to the climate system. Agricultural and Forest Meteorology 169: 156–173.

[aps311338-bib-0084] Rivera, G. , and R. Borchert . 2001 Induction of flowering in tropical trees by a 30‐min reduction in photoperiod: Evidence from field observations and herbarium specimens. Tree Physiology 21: 201–212. 11276414 10.1093/treephys/21.4.201

[aps311338-bib-0085] Rossi, S. , and N. Isabel . 2016 Bud break responds more strongly to daytime than nighttime temperature under asymmetric experimental warming. Global Change Biology 23: 446–454. 27196979 10.1111/gcb.13360

[aps311338-bib-0086] Soltis, P. S. 2017 Digitization of herbaria enables novel research. American Journal of Botany 104: 1281–1284. 29885238 10.3732/ajb.1700281

[aps311338-bib-0087] Sorensen P. O. , P. H. Templer , and A. C. Finzi . 2016 Contrasting effects of winter snowpack and soil frost on growing season microbial biomass and enzyme activity in two mixed‐hardwood forests. Biogeochemistry 128: 141–154.

[aps311338-bib-0088] Stucky, B. J. , R. Guralnick , J. Deck , E. G. Denny , K. Bolmgren , and R. Walls . 2018 The plant phenology ontology: A new informatics resource for large‐scale integration of plant phenology data. Frontiers in Plant Science 9: 517. 29765382 10.3389/fpls.2018.00517 PMC5938398

[aps311338-bib-0089] Taylor, S. D. 2019 Estimating flowering transition dates from status‐based phenological observations: A test of methods. PeerJ 7: e7720. 31579602 10.7717/peerj.7720 PMC6764363

[aps311338-bib-0090] Taylor, S. D. , J. M. Meiners , K. Riemer , M. C. Orr , and E. P. White . 2019 Comparison of large‐scale citizen science data and long‐term study data for phenology modeling. Ecology 100: e02568. 30499218 10.1002/ecy.2568 PMC7378950

[aps311338-bib-0091] Theobald, E. J. , I. Breckheimer , and J. HilleRisLambers . 2017 Climate drives phenological reassembly of a mountain wildflower meadow community. Ecology 98: 2799–2812. 29023677 10.1002/ecy.1996

[aps311338-bib-0092] Thuiller, W. , C. Albert , M. B. Araujo , P. M. Berry , M. Cabeza , A. Guisan , T. Hickler , et al. 2008 Predicting global change impacts on plant species’ distributions: Future challenges. Perspectives in Plant Ecology, Evolution and Systematics 9: 137–152.

[aps311338-bib-0093] USA National Phenology Network . 2018 Annual report: Taking the pulse of our planet. Website https://www.usanpn.org/files/npn/reports/USA-NPN-AnnualReport2018.pdf [accessed 18 March 2020].

[aps311338-bib-0094] Viskari, T. , B. Hardiman , A. R. Desai , and M. C. Dietze . 2015 Model‐data assimilation of multiple phenological observations to constrain and predict leaf area index. Ecological Applications 25: 546–558. 26263674 10.1890/14-0497.1

[aps311338-bib-0095] Vitasse, Y. 2013 Ontogenic changes rather than difference in temperature cause understory trees to leaf out earlier. New Phytologist 198: 149–155. 23347086 10.1111/nph.12130

[aps311338-bib-0096] Wadgymar, S. M. , R. M. Mactavish , and J. T. Anderson . 2018a Transgenerational and within‐generation plasticity in response to climate change: Insights from a manipulative field experiment across an elevational gradient. American Naturalist 192: 698–714. 10.1086/700097 30444658

[aps311338-bib-0097] Wadgymar, S. M. , J. E. Ogilvie , D. W. Inouye , A. E. Weis , and J. T. Anderson . 2018b Phenological responses to multiple environmental drivers under climate change: Insights from a long‐term observational study and a manipulative field experiment. New Phytologist 218(2): 517–529. 29451307 10.1111/nph.15029

[aps311338-bib-0098] Walther, G. R. , E. Post , P. Convey , A. Menzel , C. Parmesan , T. J. C. Beebee , J. M. Fromentin , et al. 2002 Ecological responses to recent climate change. Nature 416: 389–395. 11919621 10.1038/416389a

[aps311338-bib-0099] Welshofer, K. B. , P. L. Zarnetske , N. K. Lany , and L. A. E. Thompson . 2017 Open‐top chambers for temperature manipulation in taller‐stature plant communities. Methods in Ecology and Evolution 69: 491–496.

[aps311338-bib-0100] Wheatley, C. J. , C. M. Beale , R. B. Bradbury , J. W. Pearce‐Higgins , R. Critchlow , and C. D. Thomas . 2017 Climate change vulnerability for species—Assessing the assessments. Global Change Biology 23: 3704–3715. 28660715 10.1111/gcb.13759

[aps311338-bib-0101] Wiggins, A. 2013 Free as in puppies: Compensating for ICT constraints in citizen science. In Proceedings of the 2013 conference on computer‐supported cooperative work, 1469–1480. Association for Computing Machinery, New York, New York, USA.

[aps311338-bib-0102] Willis, C. G. , B. Ruhfel , R. B. Primack , A. J. Miller‐Rushing , and C. C. Davis . 2008 Phylogenetic patterns of species loss in Thoreau's woods are driven by climate change. Proceedings of the National Academy of Sciences USA 105: 7029–17033. 10.1073/pnas.0806446105 PMC2573948 18955707

[aps311338-bib-0103] Willis, C. G. , E. R. Ellwood , R. B. Primack , C. C. Davis , K. D. Pearson , A. S. Gallinat , J. M. Yost , et al. 2017 Old plants, new tricks: Phenological research using herbarium specimens. Trends in Ecology and Evolution 32: 531–546. 28465044 10.1016/j.tree.2017.03.015

[aps311338-bib-0104] Wolkovich, E. M. , and E. E. Cleland . 2011 The phenology of plant invasions: A community ecology perspective. Frontiers in Ecology and the Environment 9: 287–294.

[aps311338-bib-0105] Wolkovich, E. M. , B. I. Cook , J. M. Allen , T. M. Crimmins , J. L. Betancourt , S. E. Travers , S. Pau , et al. 2012 Warming experiments underpredict plant phenological responses to climate change. Nature 485: 494–497. 22622576 10.1038/nature11014

[aps311338-bib-0106] Yang, L. H. , and V. H. W. Rudolf . 2010 Phenology, ontogeny and the effects of climate change on the timing of species interactions. Ecology Letters 13: 1–10. 19930396 10.1111/j.1461-0248.2009.01402.x

[aps311338-bib-0107] Yost, J. M. , P. W. Sweeney , E. Gilbert , G. Nelson , R. Guralnick , A. S. Gallinat , E. R. Ellwood , et al. 2018 Digitization protocol for scoring reproductive phenology from herbarium specimens of seed plants. Applications in Plant Sciences 6(2): e1022. 29732253 10.1002/aps3.1022 PMC5851559

[aps311338-bib-0108] Yue, X. , N. Unger , T. F. Keenan , X. Zhang , and C. S. Vogel . 2015 Probing the past 30‐year phenology trend of US deciduous forests. Biogeosciences 12: 4693–4709.

[aps311338-bib-0109] Zalamea, P. C. , F. Munoz , P. R. Stevenson , C. E. Timothy Paine , C. Sarmiento , D. Sabatier , and P. Heuret . 2011 Continental‐scale patterns of *Cecropia* reproductive phenology: Evidence from herbarium specimens. Proceedings of the Royal Society B, Biological Sciences 278: 2437–2445. 10.1098/rspb.2010.2259 PMC3125618 21227965

[aps311338-bib-0110] Zipf, L. , and R. B. Primack . 2017 Humidity does not appear to trigger leaf out in woody plants. International Journal of Biometeorology 61: 2213–2216. 28828598 10.1007/s00484-017-1428-8

